# Commentary: Rare earth elements affect the growth and fitness of free-floating plant *Lemna minor* L

**DOI:** 10.3389/fpls.2026.1805616

**Published:** 2026-03-10

**Authors:** Sándor Szabó, Gergő Koleszár

**Affiliations:** Department of Biology, Institute of Environmental and Natural Sciences, University of Nyíregyháza, Nyíregyháza, Hungary

**Keywords:** *Lemna*, precipitation, rare earth elements, relative growth rate, toxicity

## 1 Introduction

The study by Gjata et al. (2025) revealed the toxicological impact of rare earth elements (REEs) on the free-floating species *Lemna minor* where they measured the effects of REEs on growth rate, photosynthetic pigment concentrations, and oxidative stress markers of the plants. A key point of such toxicity studies is the precise measurement of the relative growth rate (RGR) of the plants which is the primary metric for determining inhibitory thresholds (Naumann et al., 2007). The authors correctly described RGR by the formula: RGR=(ln(N_t_)−ln(N_0_))/t, where *N*_0_​ and *N*_t_​ are the initial and final frond numbers, and *t* is time in days. However, examination of the RGR values in their **Table 1** reveals great mathematical and biological contradiction. This commentary reveals the error source, recalculates growth rates, and discusses suggestions for the study’s conclusions on REE toxicity.

**Table 1:** Re-analysis of control group growth data from Gjata et al. (2025).

Time (days)	Reported Value* (Table 1)	Derived Nt (fronds)**	Corrected RGR (day^-1^)
3	6.5	59.5	0.132
7	9.52	106.6	0.140
12	10.05	160.6	0.116

*Reported Value = (N_t_−40)/t.

**Recalculated number of fronds.

## 2 Analysis of the reported growth data

### 2.1 Biological unlikelihood of RGR values

Under optimal conditions, typical RGRs for duckweed range from 0.15 to 0.5 day^-1^ (Ziegler et al., 2015). Hence, the RGR values reported in **Table 1** (Gjata et al., 2025) exceed the biologically reasonable rates for *L. minor* by one order of magnitude. As an example, the mean control RGR for day 7 was indicated as 9.52 day^-1^. With an initial frond number of N_0_ = 10, this rate would result in a final frond number (Nt​) over 7 days (t=7) as follows: Nt=N_0_·e^(RGR×t)^ = 10·e^(9.52×7)^ ≈ 8.74×10^29^ fronds—an astronomical and impossible figure.

### 2.2 Misuse of the growth formula

The pattern of reported values suggests that the authors calculated the absolute increase in frond number per day, that is (*Nt*​−*N*0​)/t instead of RGR.

Using the reported day 7 control RGR (9.52), and an initial N_0_ = 40 fronds (Section 2.1), we derive Nt=106.6 fronds. Applying the correct formula gives RGR = 0.140 day^-1^, a biologically realistic value. Recalculating all control time points in a similar way would yield us plausible RGRs between 0.116 and 0.140 day^-1^ (**Table 1**), consistent with nutrient or space limitations in 15 mL medium.

### 2.3 Irregular repetition of numerical values

Beyond the miscalculation, specific values appear identically across different treatments:

9.52 was reported for control (7 days), neodymium (1 mM, 7 days), and gadolinium (0.1 mM, 7 days). 10.05 appears for control (12 days), neodymium (1 mM, 12 days), and gadolinium (0.1 mM, 12 days). The probability of identical means (to two decimals) from independent biological replicates is negligible. This strongly suggests a compilation error in **Table 1** (Gjata et al., 2025) —likely inadvertent copying of control values—undermining the reliability of growth data for Nd and Gd treatments.

### 2.4 Contradictions between visual and quantitative data

Visual observations (Figure 1) also contradict the reported quantitative metrics in some instances. For cerium at 0.1 mM, apparent frond inhibition in images contrasts with the reported growth stimulation and chlorophyll increase. For neodymium, the reported superior growth at 1 mM compared to 0.1 mM conflicts with both the visual phenotype (reduced growth) and lower chlorophyll concentration at the higher concentration.

### 2.5 Methodological errors

The growth medium contained 0.485 mM phosphate (66 mg L^-1^ KH_2_PO_4_). Trivalent REE ions form insoluble phosphate precipitates (K_sp_ 10^-25^–10^-28^; Firsching and Brune, 1991). Adding nominal REE doses (0.1–1 mM) would, thus, precipitate a significant fraction, drastically reducing the bioavailable REE^3+^ this way. Although the authors note this may “mask the actual toxicity” (Section 2.2), they still proceeded with the unreasonably phosphate-rich medium. Consequently, the reported toxicity results lead to substantial underestimation of true REE toxicity and invalidate the environmental relevance of dose-response data.

In order to get environmentally relevant toxicity data, dose-response curves must be constructed and effective dose concentrations must be calculated by fitting a suitable equation. From two concentrations (0.1 mM, 1 mM) such conclusions are not possible.

## 3 Discussion

The identified flaws, altogether, compromise the study’s conclusions:

The incorrect RGR calculation undermines quantitative growth comparisons central to assessing hormesis (e.g., Ce stimulation) and inhibition. Recalculated RGRs (**Table 1**) would fall within the expected range, but the original values distort dose-response relationships.

Repeated numerical values in **Table 1**. likely indicate possible data compilation errors, particularly for Nd and Gd treatments. This raises concerns about the validity of the primary dataset and requires re-evaluation from the basic data.

Phosphate precipitation is a fundamental methodological error, meaning that the nominal REE concentrations did not reflect the bioavailability. This leads to an underestimation of toxicity and strongly reduces the relevance of the results for environmental risk assessment.

To ensure reliability, hence, we recommend recalculation of all RGR values using the correct formula and clarification of the anomalously repeated values in **Table 1**.

## 4 Conclusion

In the study by Gjata et al. (2025) critical errors in growth rate calculation, evidence of data compilation irregularities, and a methodological error involving phosphate precipitation collectively undermine the reliability of the reported toxicity thresholds. Correcting these issues would be essential for the accurate interpretation and application of the findings in environmental risk assessment.
